# Attachment, suicidal behavior, and self-harm in childhood and adolescence: a study of a cohort of Brazilian schoolchildren

**DOI:** 10.1186/s12887-023-04215-7

**Published:** 2023-08-17

**Authors:** Orli Carvalho da Silva Filho, Joviana Quintes Avanci, Thiago de Oliveira Pires, Raquel de Vasconcellos Carvalhaes Oliveira, Simone Gonçalves Assis

**Affiliations:** 1 National School of Public Health / Oswaldo Cruz Foundation (Ensp/Fiocruz), Rio de Janeiro, Brazil; 2 National Institute of Infectious Diseases/ Oswaldo Cruz Foundation (NIID/Fiocruz), Rio de Janeiro, Brazil

**Keywords:** Suicide ideation, Suicide attempt, Self-harm, Suicide, Attachment, Children, Adolescent

## Abstract

**Background:**

Attachment influences the development and the formation of the self and subjectivity and, just as early adverse events, may be related to the occurrence of mental disorders, suicidal behavior, and self-harm throughout life. This study aimed to analyze the effect of mental representation of attachment in children on suicidal behavior and self-harm throughout childhood and adolescence, considering the mediating role of internalizing problems.

**Methods:**

Based on a cohort of 500 students (mean age 8 years, SD 1.2) sampled from public schools in a Brazilian southeastern metropolis, 316 children were followed for eight years in three waves (2006, 2008, 2012). The following data from the research baseline (2005) were used: family drawing, maternal and family variables, and sociodemographic data. The mental representation of attachment (independent variable) was measured by the Family Drawing Global Scale, discriminating between secure attachment and non-secure attachment. Suicidal behavior/self-harm (dependent variable) and internalizing problems were evaluated in three research waves through CBCL and YSR (ASEBA). Descriptive analysis, calculation of frequencies and p-values of the variables of interest, as well as modeling of structural equations, were performed.

**Results:**

The prevalence throughout the study was: 17.1% [CI 13.3–20.8] for suicidal ideation and 8.9% [CI 5.6 – 12.2] for self-harm; there was a recurrence at one time in 16.5% [IC 12.6 – 20.3] and in two or more moments in 4.1% [CI 2.0 – 6.3] of the sample. Female gender (*p* = 0.035), internalizing disorders (*p* < 0.01), and non-secure attachment (*p* = 0.035) were associated with the occurrence of suicidal behavior/self-harm. The modeling indicated that 92,2% of the total effect of attachment (*p* = 0.069) on suicidal behavior/self-harm was due to direct effect, the other 7,8% of the effect being mediated by internalizing problems, adjusted for the confounding variables sex, skin color/race, and social stratum. The total effect showed a positive value, which indicates an increase in suicidal behavior/self-harm when the non-secure attachment is present. The approximate OR of non-secure attachment on the total effect (direct + indirect) was 1.15, indicating that, when adjusting for confounding variables, there was a 15% increase in suicidal behavior/self-injury from non-secure attachment.

**Conclusions:**

The study supports the hypothesis that there is a relationship between disruptive attachment patterns (non-secure attachment) developed during infancy and suicidal and self-harm behavior during childhood and adolescence. These findings validate the concern about the first thousand days of childhood as a critical period for child growth and development, but also for the mental health of children and adolescents.

## Background

Attachment is an affective bond in which an individual's sense of security is strongly linked to their attachment figure, providing relational comfort and stability [1–3]. From the perspective of child development, attachment presents a specific direction: from the child to their primary caregiver, a responsible person who becomes a secure emotional base for the infant's environmental and relational exploration and, therefore, for the species-specific developmental trajectory [1, 2, 4, 5]. In the initial propositions of Attachment Theory, Bowlby described how the effects of affective deprivation and violence predispose children to the emergence of deviations in the formation of attachment with important repercussions in the short-term (physical—failure to thrive—and developmental delays—gross and fine motor, language, social and cognitive skills) and medium-term (antisocial behavior and relational problems) [1, 6, 7]. This theoretical framework has been valued in research on the influence of early adverse events on children and adolescents’ mental health, with emphasis on suicidal and self-harm behavior [8–13].

On the premise that the attachment pattern built in early childhood influences the formation of the self and subjectivity and that it may reflect how subjects explain themselves (differentiating yourself from others ones—*self*) and their relationship with the social world (as an expression of your subjectivity), the expansion of the investigation of attachment beyond infancy became relevant [14–18]. This became possible when the empirical observation of the attachment-building process in early childhood [2, 19] was expanded to a retrospective investigation of its mental representation [14]. This shift, from observational to representative, encouraged the use of different forms of language for identification and research on attachment.

In this context, various instruments and techniques were developed to assess attachment, through mental representation, in different age groups [5, 14, 20]. On the basis that children appreciate the act of drawing and because the drawings of children up to 7 or 8 years of age express their thinking and affection, Fury [5, 21], following Kaplan and Main, proposed scales for measuring the mental representation of attachment based on drawings by schoolchildren [5, 21, 22]. Thus, based on the family drawing technique, it became methodologically possible to measure the mental representation of attachment, allowing for longitudinal assessments of its influence on mental health among children and adolescents [5, 21, 22].

The academic importance given to Attachment Theory was not linear and more recent dialogues with neurobiology and epigenetics have allowed for a new arsenal of arguments for this theory [23–25]. This adds to the relevance that the gestational context, affective and relational investment, and stimulation directed at the first thousand days of childhood assume as important variables not only for infant survival and growth but also for neurodevelopment and its repercussions on mental health [26–31].

Investigating adults who survived abuse and living in shelters, Touati et al. [9] corroborate the idea that adverse events and contexts experienced by infants during a sensitive period of their lives can disruptively interrupt neurobiological development, negatively impacting the construction of attachment bonds, increasing the risk of mental disorders, suicidal behavior, and self-harm. Being less adaptable to the subject and to their social group, insecure and disorganized attachment patterns facilitate the formation of an anomalous self and the emergence and crystallization of behavioral deviations, negatively influencing emotional regulation, a psychopathological condition related to self-harm with or without suicide intention. As Adam argues, suicidal behavior is an extreme expression of attachment [18]. These arguments are close to the conclusions of the systematic review conducted by Zortea and O'Connor [32]: insecure attachment acts as an emotional vulnerability in the face of lifelong adversity, reducing coping capacity and increasing the risk of suicidal and self-harm behavior.

Suicide is one of the most drastic outcomes in mental health and it is strongly associated with emotional distress and mental disorders, especially with internalizing problems such as anxiety and depression [23, 33–36]. It is the final phase of *a continuum* involving ideation, planning, threats, and suicide attempts, in multiple contexts where there may be overlapping self-harm without suicidal intent [33, 37, 38]. As a fatal act of this behavior, more specifically measured by the direct and indirect impacts on the health of global youth, suicide, and mental disorders have received increasing attention from pediatrics and international organizations [38–41].

Even if we consider variations in mortality rates in different countries, suicide stands out globally as the fourth cause of death among young people aged 15 to 29 [40]. Considering adolescence, 88% of suicides occur in underdeveloped or developing countries, increasing the challenges faced by those countries in their approach to public health [40].

There is no consensus in the literature regarding the definition or nomenclature of the polymorphic phenomenon of self-harm, even though important attempts to classify it as self-harm with or without suicide intention have been recognized [42–45]. Even though a structured assessment can psychopathologically differentiate between those two groups [38, 42, 46], national and global statistics sometimes group suicide attempts and non-suicidal self-harm [40, 41, 47]. Rather than only seeking this distinction, categorizing it as belonging to the suicide *continuum* or not, or evaluating it as using a lethal or non-lethal method [37, 48], it is certain that self-harm expresses, to some degree, psychological distress in children, adolescents, and young people [23, 43]. Conceptual dissensions make it difficult to accurately assess the occurrence of self-harm during adolescence, with estimates indicating their lifetime prevalence at 18% [45]. As with suicide, there is a greater occurrence of self-harm starting in the second half of adolescence, bringing them closer, in theory, to emotional distress and suicidal behavior [38, 44, 45]. As an initial phase of the suicide *continuum*, there is an increased occurrence of suicidal ideation in adolescence; although more difficult to be measured, Lima et al. (2020) [49] demonstrated an alarming trend of its increase in Brazilian adolescents between the years 2006 and 2016.

To reduce the occurrence of suicidal behavior and self-harm among young people in the world, a global effort has been made to understand these complex and multidetermined phenomena, as well as to identify their—similar and distinct—early factors of risk and prevention [39, 40, 50]. Strategies that consider developmental, relational, cultural, and social specificities of different age groups are more sensitive and effective [23, 33, 40, 43]. Thus, expanding the understanding of elements and experiences that may influence the mental health of children and adolescents at an early age becomes relevant, especially when global data suggest that 75% of mental disorders in adults begin in childhood and adolescence, 50% of which up to the age of 14 [51]. A greater understanding of the evolutionary trajectory of disruptive attachment in childhood and its relationship with emotional dysregulation, mental disorders, and suicidal behavior fills existing academic gaps and contributes to pediatric mental health care strategies.

Thus, because of the premise that attachment is a core element in child and adolescent development and based on the hypothesis that non-secure attachment plays an important role in the occurrence of suicidal behavior and self-harm, this study aimed to analyze the effect of mental representation of attachment in children on suicidal behavior and self-harm throughout childhood and adolescence, considering the mediating role of internalizing problems.

## Methods

This is a prospective cohort study with students selected in 2005, focusing on mental health and violence, in a municipality with just over 1 million inhabitants in a metropolitan region of Southeastern Brazil, characterized by high social inequality, poor infrastructure, and high levels of poverty and violence [52–54]. This article is based on baseline data obtained in 2005 and the follow-up data obtained in 2006, 2008, and 2012, covering the period between childhood and adolescence in the cohort.

### Study population

The sample plan for this study began in 2005 with children from public schools in a municipality and was based on students in the second year of elementary school (6,589 students). Cluster sampling was used with three stages of selection: schools (proportional to size), classes (simple random), and students per class (simple random) totaling 500 students (proportion of 50%, confidence level of 98.02%, and relative error of 5%). In 2006, 2008, and 2012 there were losses of 5.6%, 10.6%, and 21.8% of the participants, respectively.

In 2005, children were evaluated at the baseline for intelligence measurement using the Wechsler Intelligence Scale (WISC III) [55] validated in Brazil and required to draw their families [5, 21, 22]. Only in 2008 and 2012 did they answer questionnaires. The adults responsible for the children, in turn, answered survey questionnaires in 2005, 2006, and 2008.

The application of WISC III and family drawing was carried out by researchers who were psychologists. After the child's initial approach and application of WISC-III, they were invited to draw their family on white A4 paper with black pencils, explaining their drawing to the observant professionals [22]. The answers to the questionnaires applied to adults responsible for the children in 2005, 2006, and 2008, and to the children/adolescents in 2008 and 2012, were obtained from interviews conducted by the research team in the schools themselves (baseline) or households.

The analyses in this article excluded: children with intellectual disabilities or those not evaluated through the WISC-III in 2005; children who did not complete the drawing in 2005; participants whose questionnaires were incomplete at some point during the study. The option to exclude intellectually disabled children was justified by the difficulty of assessing behavioral problems in those children through the application of questionnaires. Of the original sample from the cohort of 500 participants, 316 participated at all points in time, being included in the study. The main reasons for the loss of individuals in the longitudinal follow-up were changes in school and city of residence over the years. The analysis of missing data in the years 2006, 2008 and 2012 showed that the profile of those who remained in the study is similar to those who started it in terms of gender, age and skin color.

### Instruments and variables

The dependent variable of interest is suicidal behavior/self-harm, measured by the presence in one or more years (2006, 2008 and/or 2012) of “non-suicidal self-injury/suicide attempt” and/or “suicidal ideation” in one or more years (2006, 2008, and/or 2012), according to caregivers or the child/adolescent themselves. This measurement was obtained using the *Child Behavior Checklist* instrument — CBCL, applied to caregivers, and *Youth Self Report* — YSR, applied to children/adolescents. The CBCL and YSR scales make up the *Achenbach System of Empirically Based Assessment* (ASEBA) [56], which is used to investigate social competence and behavior problems. Two ASEBA questions were used to assess the presence of the outcome: “deliberately try to hurt or kill myself” (non-suicide self-injury/suicide attempt) and “think about killing myself” (suicidal ideation).

The questions about non-suicidal self-injury/suicide attempt and suicidal ideation were grouped under the construct “suicidal behavior/self-harm” based on the low prevalence of the studied phenomena; because these constructs somehow address the same interest group; and because the participants were evaluated in the period between childhood and the first half of adolescence, a time when the boundaries between the studied problems are not always easy to define.

The independent variable of interest is the mental representation of attachment, measured according to the family drawing drawn by the children at the baseline of the research. The technique for evaluating the mental representation of attachment based on the child's family drawing was conceived by Kaplan & Main (1985) [14] and refined by Fury (1996, 1997) [5, 21]. Each drawing was evaluated based on the Global Rating Scale of Attachment Relationship [5, 22], consisting of eight subscales: *(i) vitality/creativity and (ii) family pride/happiness,* which relate to the feeling of security and are used to assess secure attachment; *(iii) vulnerability*, which relates to anxious-resistant/ambivalent attachment; (*iv) role-reversal,* which relates to anxious-resistant attachment*; (v) role-reversal,* which concerns anxious-resistant attachment; *(v) role-reversal) emotional distance/isolation,* which relates to anxious attachment- avoidant; *(vi) tension/anger*, a subscale related to anxious-avoidant attachment; *(vii) bizarreness/dissociation*, which evaluates disorganized-disoriented attachment; and *(viii) global pathology*, which seeks to combine the above items and chart an emotional health index for children in the context of the family, assessing feelings of insecurity in general. Each of these subscales is evaluated according to a score ranging from 7 (very high) to 1 (very low). The scores were grouped into three categories: high ('very high', 'high', 'moderately high'), moderate and low ('moderately low', 'low', and 'very low').

Each drawing was evaluated by two researchers and the disagreements were debated by a judge, who delimited the results. For analysis purposes, the category “non-secure attachment” was constituted as a group of disruptive attachment patterns, that is, the patterns diverging from secure attachment: anxious-resistant/ambivalent, anxious-resistant, disorganized-disoriented. The classification of the mental representation of attachment as “non-secure” was defined by the presence of two or more answers in the high category (score 5 to 7) among the five subscales: vulnerability, role-reversal, emotional distance/isolation, tension/anger, bizarreness/dissociation. In addition to advancing into the specificities of disruptive attachment patterns, we opted for the evolutionary understanding of this deviation in the species' trajectory about suicidal behavior. The term “non-secure” was chosen because some previous classifications used the term “insecure” to specifically group the anxious-resistant/ambivalent and anxious-resistant patterns [5, 14].

Sociodemographic variables, internalizing problems, and maternal and family variables were also included. The sociodemographic variables considered were collected at the baseline in 2005: biological sex (male or female), age, self-reported skin color (white, black, brown), and social stratum of the family, measured using the Brazil/ABEP Criterion [54], which evaluates household consumption estimated by the educational level of the parents/guardians and the accumulation of material assets in the household.

The presence of internalizing problems was measured by the ASEBA CBCL and YSR scales [56, 57] in the years 2006, 2008, and 2012, which assess symptoms of retreatment/depression, anxiety, and somatic complaints. Three groups were defined based on the T-score: non-clinical/borderline in all years (T <  = 63) and clinical in at least one point of the cohort (T > 63). The consideration of the presence of internalizing problems was justified by the association, recognized in literature, between depression and suicide and by the prevalence of depressive and anxiety disorders in childhood [10, 35]. Cronbach's Alpha for internalizing problems (CBCL, YSR) obtained at the beginning of the study was satisfactory (0.83).

**Maternal and family variables:** five variables measured in the first year of the cohort were innitialy investigated: (1) the mother's feelings during the child's pregnancy (happy, normal, and sad); (2) the pregnancy period (peaceful, disagreements, and fights); (3) breastfeeding (yes, no); (4) feeling responsible for the child (very satisfied, satisfied, dissatisfied); (5) and the involvement of the primary caregiver with the child in early childhood, following the 10-item scale proposed by Willms [58, 59]. It assesses positive interaction (praise, games, playing, talking, laughing, reading with the child) and topics such as discipline, obedience, and discredit, measured by frequency “always, many times, few times, and never”. The items were aggregated and analyzed using averages; a higher score indicates better family involvement. This scale was adapted cross-culturally in the first wave of research, with a Cronbach alpha of 0.72 [59].

### Data analysis

For the 316 participants, the presence of non-secure attachment was assessed based on the five subscales that measure this category. The descriptive analysis was performed by calculating the frequencies and p-values of variables of interest.

**Fig. 1:**
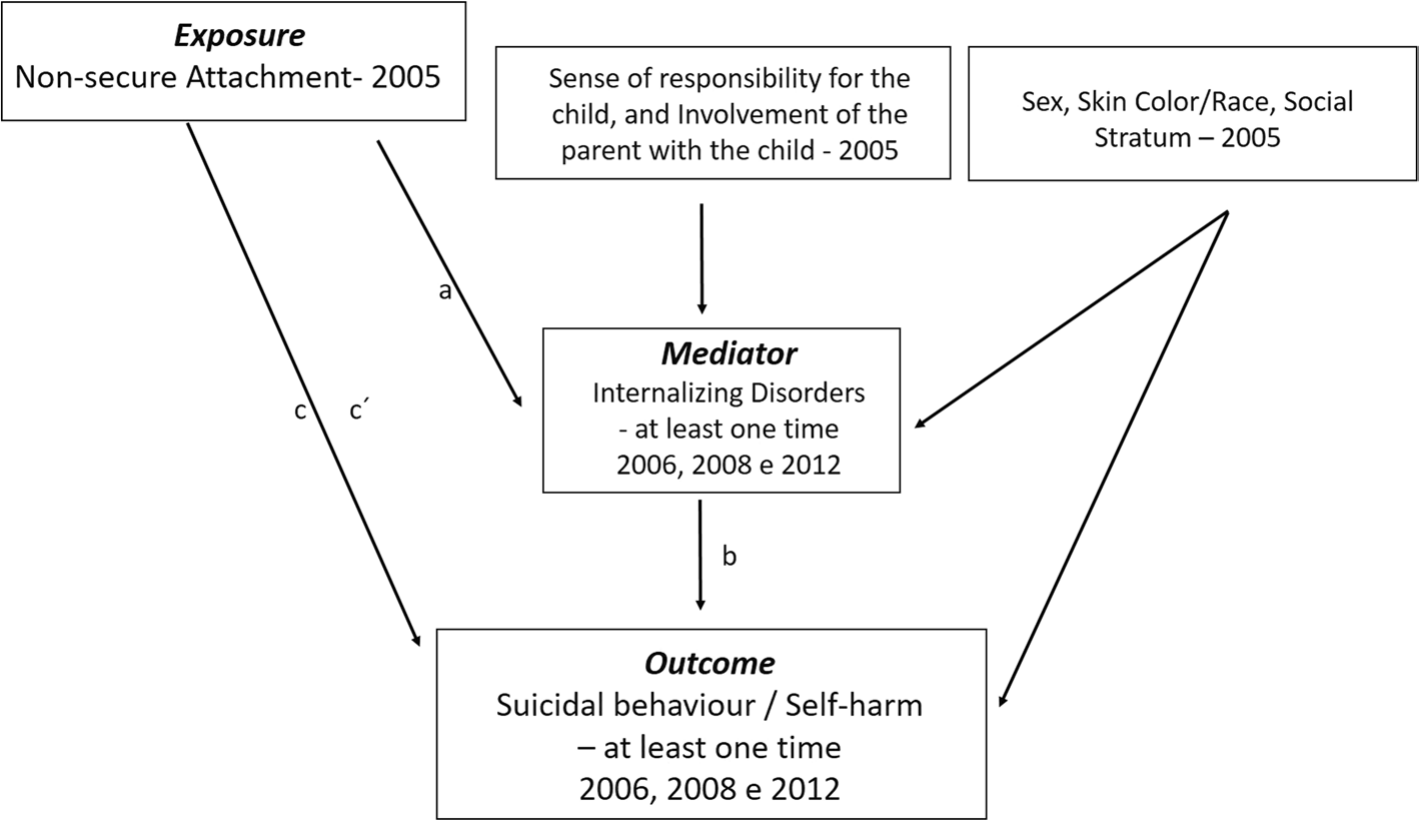
Theoretical model. Note: a = Exposure Effect—> mediator; b = Mediator Effect; c = Total Effect; c’ = ADE (Average Direct Effects); a*b = Indirect Effect or ACME (Average Causal Mediation Effects); c = c’ + a*b = ADE (Average Direct Effect) + ACME (Average Causal Mediation Effects)

Next, a structural equation model was used to assess the relationship between non-secure attachment and suicidal behavior and self-harm, as well as the mediating role of internalizing disorders. Structural equation modeling extends the analysis by examining a series of dependency relationships simultaneously. The structural model carried out used the outcome suicidal behavior/self-harm (yes/no) and is therefore a logistical model with a probit-type connection function. The model was adjusted for sex, race/color, social status, sense of responsibility for the child, and involvement of the parent with the child, potentials confounding variables. In Results section, the Fig. 1 illustrates the theoretical model of the study that guided the structural model built in the analytical phase.

Based on the theoretical model in Fig. 1 and the results of the β coefficients, other measurements were extracted such as indirect effect, direct effect, and proportion of mediation (indirect effect/total effect). The indirect effect is calculated as the product of the estimates of the attachment variable and the mediating variable (internalizing disorders). The total effect is the sum of indirect and direct effects.

Since the model does not yet allow the use of the logit link function, an approximation was used to extract the Odds Ratio (OR) from the estimates of the β coefficients:$exp(estimate\times \left(\pi /\surd 3\right))$. The fit quality of the model was considered when RMSEA < 0.05 and CFI > 0.95.

The approximate OR of insecure attachment on the total effect (direct + indirect) was calculated. The analysis of the data and results presented incorporates the expansion to the population of students from the public school system [60]. The statistical package used was R version 4.2.1 [61], the library for the model of equations structure was lavaan and the library for inclusion in the sample plan was lavaan.survey [62, 63].

## Ethical issues

All stages of the research were approved by the Research Ethics Committee of the National School of Public Health/Oswaldo Cruz Foundation (CAAE): 0051.0.031.000–04; 0024.0.031.000–07; 0040.0.031.000–08; 0071.0.031.000–11). All caregivers signed an Informed Consent Form; the adolescents signed a Consent Form to participate in the research. Cases that were clinically serious and that were not undergoing psychological or psychiatric care received clinical advice and referrals.

## Results

In 2005, 74.6% of the participants were between 6 and 8 years old, 21.4% were between 9 and 10 years old, and 4% were 11 to 13 years old (mean 8 years, SD 1.2). The eight years of follow-up of the cohort analyzed are enough to characterize the years that precede and follow the transition from childhood to adolescence.

Figure 1 shows the theoretical model that governs the analyzes carried out. It allows evaluating the effect of exposure (insecure attachment) on the suicidal behavior/self-injury total effect (direct + indirect), adjusted for the confounding variables.

**Fig. 2:**
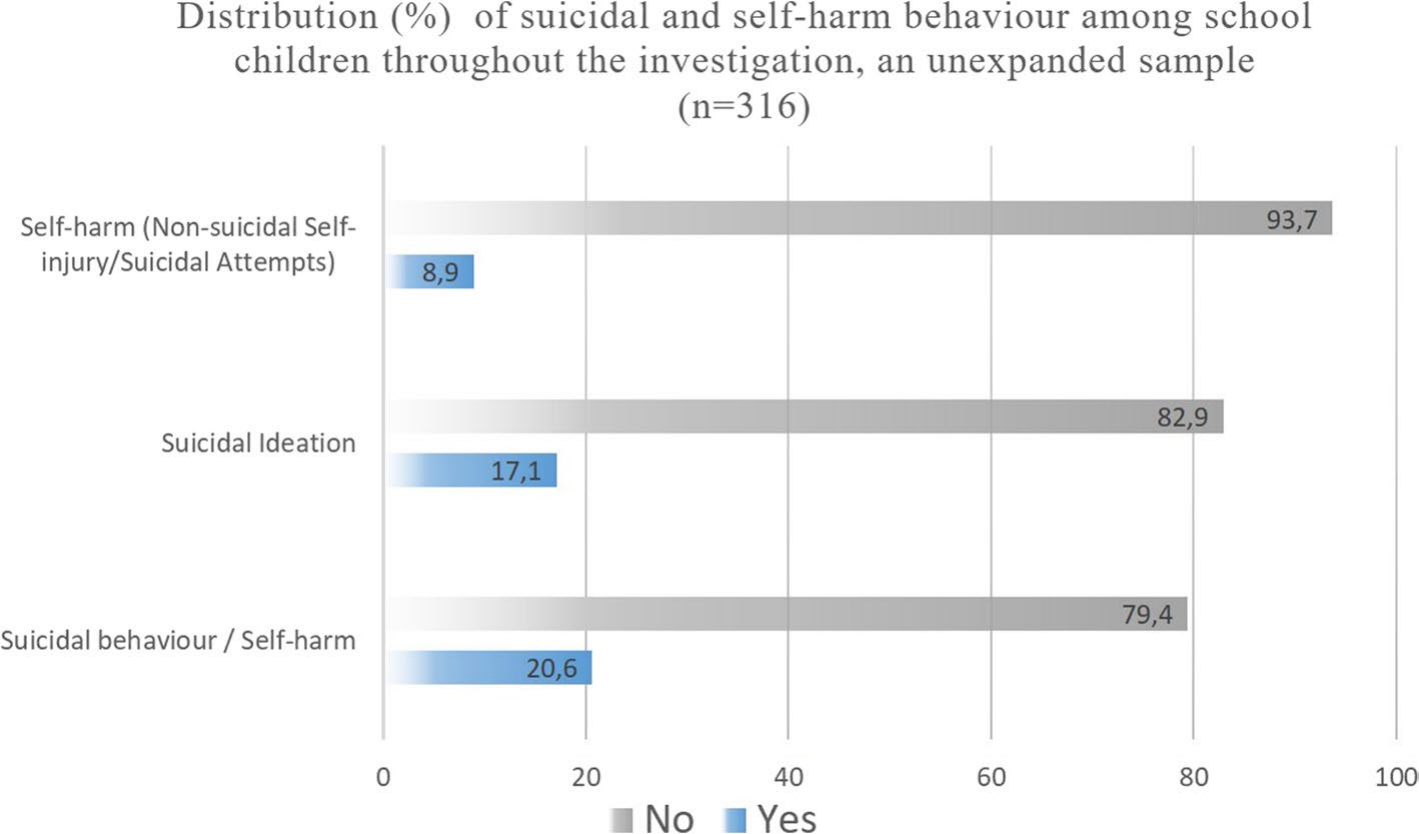
Distribution (%) of suicidal and self-harm behavior among school children throughout the investigation, an unexpanded sample (*N* = 316)

Figure 2 shows the frequency of suicidal behavior and self-harm among participants over the three research waves (2006, 2008, and 2012). Frequencies are presented for each item measured for suicidal behavior and self-harm. The items are also presented in a grouped form, allowing the visualization of the proportion of suicidal behavior/self-harm throughout the investigation: absence, presence, and recurrence.

The presence of suicidal ideation was found in 17.1% [13.3 – 20.8] of the participants during the years 2006, 2008, and 2012, and the presence of self-injuries/attempts in 8.9% [5.6 – 12.2] of them. The data show that 79.4% of the children have never experienced any manifestation of suicidal behavior or self-harm. Among the 20.6% [CI 16.4 – 24.7] with these symptoms, 16,5% [CI 12.6 – 20.3] reported it in just one year of the investigation, and 4.1% [ CI 2 – 6.3] in two or more moments.

Table 1 shows the results of the intersections of the variables with the outcome suicidal behavior/self-harm. Among the sociodemographic variables, only the female sex is associated with suicidal behavior/self-harm; age is worth mentioning because it is close to statistical significance. Skin color and social stratum showed no association with the outcome.

**Table 1:** Prevalence of suicidal behavior/self-harm according to sociodemographic, child and adolescent mental health, maternal and family variables

Variables	Categories	Suicidal behavior (%)	*p*-value
**Sociodemographic variables**			
Sex	Female (*N* = 2162)	26.6	**0.035**
	Male (*N* = 2054)	14.8	
Skin color/ethnicity	White (*N* = 1401)	14.3	0.207
	Black (*N* = 467)	25.7	
	Brown (*N* = 2294)	22.1	
Age^a^	-	Average = 8.1/DP = 1.3	0.056
Social stratum	A + B + C (*N* = 2002)	17.3	0.318
	C + D (*N* = 1521)	22.8	
**Variables on the mental health of children and adolescents**			
Internalizing disorders	Clinic (*N* = 1015)	36.8	** < 0.001**
	Normal/Borderline (*N* = 3202)	15.4	
Non-secure attachment	Yes (*N* = 1842)	24.6	**0.035**
	No (*N* = 2375)	17.4	
**Maternal and family variables**			
Mother's feeling during a child's pregnancy	Cheerful (*N* = 1468)	18.2	0.346
	Normal (*N* = 1067)	17.5	
	Sad (*N* = 960)	24.9	
Gestation period	Peaceful (*N* = 2440)	20.8	0.994
	Disagreements/fights (*N* = 1603)	20.8	
Breastfeeding	Yes (*N* = 3790)	19.7	0.281
	No (*N* = 319)	29.2	
Feelings of responsibility towards the child	Very satisfied (*N* = 3562)	17.2	**0.011**
	More or less satisfied (*N* = 359)	40.7	
	Dissatisfied (*N* = 54)	49.7	
Caregivers’ involvement with the child	Yes (*N* = 867)	Average = 27.6/DP = 4.6	0.096
	No (*N* = 3350)	Average = 28.6/DP = 4.9	

*SD* standard deviation

^a^Age — average in years

Regarding the mental health of children and adolescents, there are more internalizing and non-secure attachment disorders among children with suicidal behavior/self-harm (*p* < 0.001 and *p* = 0.035, respectively).

There was no association between suicidal and self-harm behavior during childhood and adolescence with variables that measure the child's in-uterus and breastfeeding phase. On the other hand, the variables that assess the feelings and involvement of the caregivers towards the child stood out. It appears that responsible adults who feel dissatisfied and more or less satisfied throughout the child's development stand out among participants with suicidal behavior/self-harm (*p* = 0.011).

The model carried out (Table 2, Fig. 1) shows three types of effects (total, direct, and indirect) of the non-secure attachment on the occurrence of suicidal behavior/self-harm, when adjusted for the confounding variables sex, skin color/race, and social stratum. All effects were positive, suggesting that attachment could increase the occurrence of suicidal behavior/self-harm. Although the effects (direct, indirect, and total) have p-values > 0.05, a borderline value was observed for the total effect (*p* = 0.069).

**Table 2:** Total, direct, and indirect effects of non-secure attachment on the suicidal behavior/self-harm

	Estimate	OR	*P*-value
Direct effect: Suicidal behavior/self-harm <—Attachment (a)	0.071	exp (0.071* (pi/sqrt (3))) = 1.14	0.104
Indirect effect (via internalizing behavior mediation) (b + c)	0.006	exp (0.006* (pi/sqrt (3))) = 1.01	0.546
Full effect (a + b + c)	0.077	exp (0.077* (pi/sqrt (3))) = 1.15	0.069

1) a = exposure effect—> mediator; b = mediator effect; c = total effect; c’ = ADE (average direct effects); a*b = indirect effect ou ACME (Average Causal Mediation Effects); c = c’ + a*b = ADE (average direct effect) + ACME (Average Causal Mediation Effects)

2) For the structural model, the probit connection function was used, and the effects were adjusted by the confounders: sex, race/color, and social stratum

Indirect effect: 0.006/0.077 = 7.8%

Direct effect: 0.071/0.077 = 92.2%

RMSEA < 0.001; CFI = 1,000

The first estimate derived from the model is the total effect, which includes both the direct actions of non-secure attachment on suicidal behavior/self-harm and the influence of internalizing problems as mediation (β _total_ coefficient = 0.077).

The second one is the direct effect of non-secure attachment on suicidal behavior/self-harm (β _direct_ coefficient = 0.071), which accounts for 92.2% of the total attachment effect. The third estimate measured is the indirect effect of non-secure attachment on suicidal behavior/self-harm, considering the influence (mediation) of internalizing problems (β _indirect_ coefficient = 0.006), arriving at the calculation that 7.8% of the total attachment effect on suicidal behavior/self-harm is mediated by internalizing disorder.

## Discussion

During the monitoring of the cohort of school children, it is worth highlighting the finding that, over the years, 20.6% [CI 16.4 – 24.7] of the participants presented at least one report of suicidal behavior/self-harm (suicidal ideation or non-suicidal self-injury/suicide attempt), with recurrence in 4.1% [CI 2 – 6.3] of all cases. This important finding refers to the clinical and epidemiological magnitude that illuminates the relevance of mental health in the global health of children and adolescents [39, 43, 45, 51]. Suicidal behavior and self-harm that may be reduced by psychosocial interventions on variables that translate into vulnerabilities, such as those identified in this research.

These prevalence rates were slightly lower than those found in 2020 by Chen and Kuo [64] in a group of 1035 13-year-old adolescents from Taiwan followed up for one year, with 20.3% ideation and 4.7% of suicide attempts. Although the sociocultural varieties between the two groups may justify these differences, the average age of the participants (8 *vs.*13) can be understood as a possible explanatory factor for this difference, associated with the difficulty in recognizing suicidal behavior during childhood [38, 46].

Official statistics compiled by the Brazilian Ministry of Health show, in line with global data, an increase in non-suicidal self-injury, suicide attempts, and suicide attempts during the transition from the first to the second half of adolescence [33, 38, 40, 45, 47]. Considering the Brazilian reality, between 2010 and 2019 the suicide rate increased from 0.31 to 0.67 per 100,000 in the group with ages between 5 and 14, while among adolescents aged 15 to 19 it went from 3.5 to 6.4 per 100,000 [47]. Since ideation and suicide attempt are clear risk factors for suicide [10, 23, 43, 44], the relevance of these data increases. The identification of recurrences in suicidal and self-harm behavior can be understood as an element of gravity, since it signals crystallization and a greater risk of a fatal outcome [50].

Considering the 316 participants in the cohort, there were no deaths due to suicide during the stages of investigation (2005 to 2012). The main explanation for this is the age of the participants, still in the first half of adolescence; [1] but it cannot be ruled out that other deaths, including suicide, have occurred in the missing cases—considering that there was no access to other sources of information.

The greater presence of suicidal behavior/self-harm among girls is a consensus in the literature [65, 66], especially when the outcomes evaluated exclude consummated suicide. Even though racial issues, social strata, and other social vulnerabilities constitute potential risk factors, when considering vulnerability and suicide, they were not significant in this sample.

Two variables were found to be relevant in association with suicidal behavior in the descriptive analysis: the presence of internalizing disorders and the presence of non-secure attachment. Regarding internalizing disorders, the literature is clear in showing the association between depression and suicide at all ages [20, 23, 65, 66] and the significant prevalence of depression and anxiety disorders in children and adolescents [39, 67]. With regard to attachment, Waraan et al. (2021) [10] demonstrated how insecure attachment directed to parents was associated with suicidal ideation in a clinical sample of depressed Norwegian adolescents undergoing treatment. In a study with Chinese adolescent students, Guo et al. (2021) [12] reached similar conclusions, proposing that insecure attachment to parents influences the occurrence of suicidal ideation, with roles played by anhedonia (as an important depressive symptom) and relationships between peers. The findings of Scandinavian and Chinese research, when investigating population groups in diverse contexts and very ethnically different from each other and from those in this research, point to similar conclusions about the relationships between depressive symptoms, deviant attachments, and suicidal behavior. This empirically confirms the importance with which the attachment, via emotional regulation, associates to the onset of psychiatric disorders and suicidal behavior. Even without using the attachment construct, the findings of Donath et al. (2014) converse with this study when they conclude that childhood experiences with adverse parenting styles may influence the occurrence of self-harm during adolescence [31].

The results regarding non-secure attachment are consistent with the arguments presented in this article and confirmed the research hypothesis. The greatest relevance in this study was the methodological choice to confirm this association. Most studies measure attachment retrospectively, questioning parents, adolescents, young people and adults and not children; and even when a longitudinal follow-up is achieved, when a non-infant population is at the baseline, they focus on a retrospective look at the assessment of suicidal and self-harm behavior during childhood and adolescence [9, 11, 32, 68]. Thus, despite the losses obtained in this study, the possibility of prospectively monitoring these 316 children directing the investigation to the occurrence of suicidal and self-harm behavior with attachment as exposure allowed relevant findings, which can serve as arguments for models of early clinical intervention, starting with the identification of children in greater vulnerability.

Of the maternal and family variables chosen for the investigation, “feeling responsible for the child” is relevant, with greater parental dissatisfaction associated with suicidal behavior/self-harm, since it confirms the importance of attachment for this outcome. Considering the average age of the children at the beginning of the investigation, the relational dynamics of parents with the child and their parenting styles, could help to understand the construction and repercussion of the relational attachment bond.Thus, even though the construct of this variable does not allow it to be used to directly measure attachment, it allowed for understanding about the family context where attachment was built [1, 9, 31].

Considering the model used, some general remarks are relevant. The full effect of attachment on suicidal behavior and self-harm deserves attention. In this sense, factors such as multiple adjustment and the incorporation of the sample plan of the complex sample into the analysis may have hindered the achievement of significant p-values [69].

Other fundamental aspects of the discussion are the observation of the insignificant relationship between attachment and suicidal behavior and self-harm when it involves internalizing problems, with only 7.8% of the total effect due to this internalizing disorder. On the other hand, after adjusting for confounding variables, the approximate OR of the total effect suggests 15% greater chance of suicidal behavior and self-harm among participants with a mental representation of non-secure attachment.. It should be noted that the attachment effect on suicidal behavior has been controlled by the confounders so that they would not distort the effect being analyzed. Considering the multi-causality of the suicide *continuum* [23, 33, 37], recognizing its relevance at such an early stage may facilitate the promotion of mental health throughout development, reinforcing the medium and long-term importance of care and affective investment in children's first thousand days, [27, 28] even when faced with less common outcomes in pediatric practice with schoolchildren and younger adolescents.

This study has limitations, of which we highlight the loss of participants throughout the cohort. However, it is worth underlining the value of studying with a community sample, recruited at a school environment and representative. The measurement of suicidal behavior through ASEBA deserves attention because it is not a specific instrument for this purpose. Despite the understanding that non-suicidal self-injury is not necessarily recognized as suicidal behavior, the frequent psychopathological overlap justified this methodological choice, validating the findings in the literature that self-harm (with or without suicidal ideation) is associated with adverse life experiences, insecure attachment, and developmental deviations [11, 23, 48]. It should be noted, however, that the higher occurrence of suicidal ideation (17.1%) compared to self-harm (8.9%) probably reflects the median age of the students surveyed, since the tendency would be to find rates of non-suicidal self-injury closer to those of suicidal ideation [43–45].

Another methodological choice that deserves discussion was the grouping of characteristics of insecure attachments and disorganized attachment in the category “non-secure attachment” measured by the Family Drawing Global Scale [5, 22]. Despite the risks, it was possible to understand that this categorization allowed greater precision in the identification of elements in the mental representation of attachment understood as a deviation in the development of attachment, despite reducing specificities in the typology of attachment. This reinforced the premise that disruptions in the attachment process are prospectively associated with self-harm, with and without suicidal ideation.

In this context, the longitudinal study with children and adolescents is the main strength of this study, standing out among the relevant studies that assess attachment and suicidal behavior retrospectively. It should be noted that the latest wave of research involved participants who were in the first half of adolescence, a stage that precedes an increase in the incidence of suicide globally [38, 40, 41]. Thus, it is possible to say that the data found anticipate a reality that may be found at a later stage in this investigation—which justifies the relevance of new studies with this perspective—but that it still causes clinical concern based on the correlations found presently.

## Final considerations

This study adds to recent findings that corroborate the relationship between attachment, suicidal behavior, and self-harm, considering that disruptive attachments are related to emotional dysregulation and mental disorders throughout life. The findings validate the concern about the first thousand days of childhood, a critical period for building attachment between children and their attachment figure, confirming the importance of attachment for child and adolescent mental health [26, 28, 29]. The guarantee of care and affectionate relationships are conditions that reduce deviations in developmental trajectories and can prevent self-harm and suicidal behavior. This argument may facilitate the early attention of health, education and social assistance professionals on child development and encourage the inclusion of all forms of self-harm in the list of pediatric concerns; likewise, it may become an encouragement for public policies to guarantee children's rights. The recognition of mental representations of attachment also facilitates the identification of children who become more vulnerable and incapable of dealing with peers and stressors during childhood and adolescence, leading ecologically to increasing contexts of violence and emotional distress. Further research is needed to expand the recognition of this association in other population groups and to try, even in childhood, to differentiate the paths that different types of attachment may facilitate as a risk or protection about suicidal behavior or non-suicidal self-harm. In the same way, it allows the topic of attachment to receive greater critical clinical attention, considering new diagnostic propositions with attachment as an important psychopathological nucleus.

## Data Availability

Part of the data used for the analysis of this study is already available in research reports; part of it can be available upon request from the corresponding author for the article due to privacy / ethical restrictions.
